# Antidiabetic Activity, Phytochemical Analysis, and Acute Oral Toxicity Test of Combined Ethanolic Extract of *Syzygium polyanthum* and *Muntingia calabura* Leaves

**DOI:** 10.1155/2024/3607396

**Published:** 2024-07-17

**Authors:** Agustinus Widodo, Evi Sulastri, Ihwan Ihwan, Mohamad Hadi Cahyadi, Saipul Maulana, Muhammad Sulaiman Zubair

**Affiliations:** Tadulako University, Palu, Indonesia

## Abstract

*Syzygium polyanthum* is known for its capacity to regulate blood glucose levels in individuals with diabetes, while *Muntingia calabura* leaves have a traditional history as an alternative therapy due to their antidiabetic compounds. The combination of these two plants is expected to yield more optimized antidiabetic agents. This study aims to assess the antidiabetic activity of the combined ethanolic extract of *S. polyanthum* and *M. calabura* leaves by measuring the *in vitro* inhibition of the *α*-glucosidase enzyme and the blood glucose level in streptozotocin-induced rats and to determine the phytochemical contents of total phenolics, total flavonoids, and quercetine as marker compounds. Acute oral toxicity test was also evaluated. Both plants were extracted by maceration using 96% ethanol. Various combinations of *S. polyanthum* and *M. calabura* leaves extracts (1 : 1, 2 : 1, 3 : 1, 1 : 3, and 1 : 2) were prepared. The *in vitro* test, along with the total phenolic and total flavonoid content, were measured by using UV-Vis spectrophotometry, while quercetine levels were quantified through high-performance liquid chromatography (HPLC). The *in vivo* and acute toxicity tests were performed on rats as an animal model. The findings demonstrated that the 1 : 1 combination of *S. polyanthum* and *M. calabura* leaves ethanolic extract displayed the highest enzyme inhibitory activity with IC_50_ value of 36.43 *µ*g/mL. Moreover, the combination index (CI) was found <1 that indicates the synergism effect. This combination also decreases the blood glucose level in rats after 28 days of treatments without significant difference with positive control glibenclamide (*p* > 0.005), and it had medium lethal doses (LD_50_) higher than 2000 mg/kg BW. Phytochemical analysis showed that the levels of total phenolics, total flavonoids, and quercetine were 30.81% w/w, 1.37% w/w, and 3.25 mg/g, respectively. These findings suggest the potential of combined ethanolic extracts of *S. polyanthum* and *M. calabura* leaves (1 : 1) as raw materials for herbal antidiabetic medication.

## 1. Introduction

Diabetes mellitus is a serious health issue that affects globally. According to the International Diabetes Federation, the global prevalence of diabetes was estimated to be 9.3% in 2019 and projected to rise by 2045 [1]. The chronic condition's diabetes elevates the incidence has spurred interest in alternative treatments beyond conventional synthetic drugs, which often have side effects including gastritis, diarrhea, hypoglycemia, abdominal pain, nausea, flatulence, headache, fatigue, vomiting, and pharyngitis. As a result, there has been a surge in exploring natural products as potential solutions for managing diabetes [2–4].

Medicinal plants offer natural antidiabetic properties with fewer side effects [5]. For instance, *Gymnema sylvestre*, an herb native to India and commonly used in Ayurvedic medicine, contains gymnemic acids that inhibit alpha-glucosidase activity, reducing blood sugar levels [6]. Similarly, mulberry leaves and bitter melon (*Momordica charantia*) extracts have shown promising results in glycemic control [4, 7]. Medicinal plants with analogous activity may form synergistic interactions that significantly enhance the therapeutic efficacy of treatments. This principle has been employed in traditional medicines for centuries, where combinations of herbs are used to effectively address various diseases. Moreover, recent studies have demonstrated that polyherbal extracts have also demonstrated enhanced therapeutic efficacy with minimal side effects [8, 9]. Studies have evaluated the *α*-glucosidase enzyme inhibitory activity of combination of extracts of *Oryza sativa* L. var glutinosa and *Orthosiphon aristatus*. Combinations of these extracts in a 1 : 1 ratio exhibited IC_50_ values of 67.82 *µ*g/mL [10]. Furthermore, the ethanol extracts of turmeric, ginger, and black tea water extract were tested for their *α*-glucosidase inhibition. The IC_50_ values obtained were 9.48 ± 0.05 g/mL for turmeric, 66.64 ± 0.44 g/mL for ginger, and 9.52 ± 0.25 g/mL for black tea. Among these extracts, the combination of turmeric, ginger, and black tea exhibited the highest *α*-glucosidase inhibition at 67.86 ± 0.93% [11].

Exploration of medicinal plants from Indonesia presents a promising source for alternative treatments including diabetes. Particularly, *S. polyanthum* and *M. calabura* have gained attention for their culinary uses as well as their reported therapeutic properties. *S. polyanthum* not only adds flavor to Indonesian cuisine but also shows promise as a natural remedy for managing diabetes. *S. polyanthum* leaves have long enjoyed a reputation for their beneficial effects in diabetes management [12]. Extensive research has revealed that *S. polyanthum* leaves possess compelling antidiabetic properties, including glucose-lowering effects and insulin-enhancing activity [13]. This rich history of traditional use, combined with scientific evidence, highlights *S. polyanthum* as a captivating natural resource ripe for exploration as an alternative for diabetes management. Recent research has revealed its potential therapeutic effects in combating this prevalent metabolic disorder. *S. polyanthum* leaves are rich in bioactive compounds such as flavonoids and polyphenols, which have demonstrated remarkable antidiabetic properties. These compounds have the potential to enhance glucose metabolism and improve insulin sensitivity, making *S. polyanthum* an intriguing natural product worth exploring for diabetes management [12, 14].

Another captivating plant species, *M. calabura*, has garnered attention for its potential therapeutic value in diabetes management. Apart from bearing delicious fruits, *M. calabura* also holds the potential to assist in the fight against diabetes. Studies have identified bioactive compounds like phenolics, flavonoids, and triterpenoids in the leaves and fruits of *M. calabura*, which exhibit significant antidiabetic properties. These compounds play a vital role in regulating blood glucose levels, enhancing insulin secretion, and protecting pancreatic cells from damage, positioning *M. calabura* as a promising natural product for diabetes management [15]. Similarly, *M. calabura* leaves have long been used as an alternative therapy due to their compounds with antidiabetic properties. Similarly, *M. calabura* leaves have been celebrated for their potential antidiabetic and antioxidant properties [16]. Scientific investigations have demonstrated that *M. calabura* leaf extracts exhibit remarkable glucose-lowering effects and offer protection against the oxidative stress commonly associated with diabetes. These remarkable findings further emphasize the therapeutic potential of *M. calabura* in the management of diabetes [17]. Combining these two plants is expected to provide a synergistic effect, potentially enhancing their efficacy in diabetes management. Our previous in silico experiment found that alpha-glucosidase was the target for metabolite compounds of both *S. polyanthum* and *M. calabura* [18].

The primary objective of this study was to evaluate the *in vitro* and *in vivo* antidiabetic potential of the combined ethanolic extract of *S. polyanthum* and *M. calabura* leaves. The assessment will be alpha-glucosidase activity inhibition assay and blood glucose decreasing level on streptozotocin-induced diabetic rats. Additionally, the investigation will further determine the total phenol, total flavonoid, and quercetine contents and also the acute oral toxicity test. The outcomes of this research are anticipated to enrich our comprehension of the antidiabetic attributes intrinsic to the combined plant extracts of *S. polyanthum* and *M. calabura* leaves. Moreover, the study seeks to illuminate the viability of these common Indonesian plants as valuable natural sources of antidiabetic agents. In doing so, it aims to lay the groundwork for deeper exploration and the development of natural interventions for the management of diabetes.

## 2. Materials and Methods

### 2.1. Materials

In this study, ethanol analytical grade was used as an organic solvent, along with phosphate buffer, 4-nitrophenyl *α*-D-glucopyranoside, hexamethylenetetramine (HMT), HCl, glacial acetic acid, AlCl_3_, Folin–Ciocalteu reagent, NaOH, and gallic acid as part of our experimental setup. *M. calabura* and *S. polyanthum* leaves samples were collected from Lembasada Village, South Banawa District, Donggala Regency, Central Sulawesi Province, in March 2023. Taxonomists from the Department of Biology, Faculty of Mathematics and Natural Sciences, Tadulako University, verified and authenticated the herbarium specimens of the plant samples under the identification number 218/UN28.1.28/BIO/2022.

### 2.2. Extraction

All samples were thoroughly rinsed under a continuous stream of tap water to remove debris and then gently dried. The cleaned *M. calabura* and *S. polyanthum* leaves were subsequently cut into smaller pieces, approximately 0.5 cm in length. Once cut, the samples were dried to complete dryness in a dehydrator set at 40°C. Once fully dried, the samples were coarsely ground into powder using an electrical grinder. The extraction process was carried out using 96% ethanol through the maceration technique for 3 × 24 hours per cycle at room temperature. After each maceration cycle, the mixture was filtered and the filtrates were evaporated by using a rotary evaporator to obtain a viscous extract.

### 2.3. *α*-Glucosidase Enzymatic Assay

The *α*-glucosidase enzyme inhibition activities were assessed employing a modified method derived from a prior study [19]. In the *α*-glucosidase assay, acarbose used as the standard drug, and the reaction mixture was carefully prepared as follows: 50 *µ*L of 0.1 M phosphate buffer (pH 7.0), 25 *µ*L of 0.5 mM 4-nitrophenyl *α*-D-glucopyranoside (dissolved in 0.1 M phosphate buffer, pH 7.0), 10 *µ*L of each test sample (at concentrations of 62.5, 125, 250, 500, and 1000 *µ*g/mL), and 25 *µ*L of *α*-glucosidase solution. To prepare the *α*-glucosidase solution, a stock solution of 1 mg/mL was diluted in 0.01 M phosphate buffer (pH 7.0) to yield a final concentration of 0.04 Units/mL, just before the assay. Following meticulous assembly, the reaction mixture underwent a 37°C incubation for 30 minutes. Termination of the reaction was accomplished by introducing 100 *µ*L of 0.2 M sodium carbonate solution. The enzymatic hydrolysis of the substrate was gauged by monitoring the liberation of p-nitrophenol within the reaction mixture, assessed at 410 nm utilizing a microplate reader. The IC_50_ calculation was based on linear regression analysis by plotting the value of sample concentrations (X) and the percentage of inhibition (Y). The combination index was calculated by using CompuSyn Software [20], employing the following equations to categorize the extract interactions: synergistic (CI ≤ 1), additive (CI = 1), and antagonistic (CI > 1).

(1) $$\begin{array}{c} \text{CI}=\frac{\left(D_{1}\right)}{{\left(Dx\right)}_{1}}+\frac{\left(D_{2}\right)}{{\left(Dx\right)}_{2}}. \end{array}$$

At this point, (*D*_1_) and (*D*_2_) represent the combination doses of *Syzygium polyanthum* extract and *Muntingia calabura* extract, respectively, that give an IC_50_ effect. Moreover, (*Dx*)_1_ and (*Dx*)_2_ reflect the single doses of *Syzygium polyanthum* extract and *Muntingia calabura* extract that elicit the same effect [21].

### 2.4. Experimental Animals

Male Wistar rats 3-4 months old weighing 200–230 g were selected for this study. They were fed with standard rats' pellet, and water was given ad libitum. The animals were acclimatized for 1 week before the induction of experimental diabetes. The experimental protocols were conducted in accordance with ethical guidelines as approved by the Ethics Committee for Medical and Health Research, Faculty of Medicine, Tadulako University (932/UN.28.1.30/KL/2023).

### 2.5. Induction of Diabetes Mellitus

Experimental diabetes was induced in overnight fasted experimental rats by a single intraperitoneal injection of streptozotocin (30 mg/kg body weight) dissolved in 0.1 M freshly prepared cold citrate buffer pH 4.5. After 36 h for development of diabetes, blood glucose was measured and rats with fasting serum glucose levels more than 200 mg/dL were considered diabetic and selected for further study.

### 2.6. Experimental Design

After the successful induction of experimental diabetes, the rats were divided into six groups each containing a minimum of 5 rats. Group 1 was given a suspension of *M. calabura* leaf extract at a dose of 200 mg/kg BW. Group 2 was given a suspension of *S. polyanthum* leaf extract at a dose of 200 mg/kg BW, and Group 3 was given a suspension of combined extracts of *M. calabura* and *S. polyanthum* leaves at a dose of 1 : 1 (200 : 200 mg/kg BW). Group 4 was given a suspension of glibenclamide 0.45 mg/kg BW, Group 5 (negative control) was given 0.5% Na-CMC, and Group 6 was a normal control without any treatment. Body weight and plasma glucose level measurements were conducted weekly during the experiment. The plasma glucose level was measured using a one-touch glucometer. The dosage of the extracts was adjusted every week to accommodate changes in body weight to maintain the same dosage throughout the experiment. They were administered orally for 28 days. After 28 days, the rats were fasted overnight and euthanized under anesthesia (ketamine) following blood sample collection and the organs of heart and kidney were dissected and stored at −20°C.

### 2.7. Acute Oral Toxicity Test

Acute oral toxicity study was performed for the combined extracts of *S. polyanthum* and *M. calabura* (1 : 1) according to the guidelines of the Organisation for Economic Cooperation and Development (OECD) [22]. The rats were kept on fasting overnight, being provided only water prior to oral dosing. Then, the extract was administered orally at different dose levels, that is, 175, 550, and 2000 mg/kg of body weight. The rats were closely observed for specific toxicities and behavioral alterations such as restlessness, tremors, diarrhea, sluggishness, weight loss, and paralysis at consistent intervals during the initial four hours following the administration of the extract for a total of 24 hours. Subsequently, daily observations were conducted over a two-week period to detect any changes in overall behavior and physical activities. Food access was provided four hours after administering the extracts [23].

### 2.8. Determination of Total Flavonoid

The quantification of flavonoid content involved the utilization of a reagent comprising multiple components: HMT (0.5% w/v hexamethylenetetramine solution), HCl (25% w/v hydrochloric acid solution), glacial acetic acid (5% v/v glacial acetic acid solution in methanol), and AlCl_3_ (2% w/v AlCl_3_ solution in glacial acetic acid solution). In the preparation of the stock solution, an extract equivalent to 200 mg of the crude simplicia was combined with 1 mL of HMT solution, 20 mL of acetone, and 2 mL of HCl solution. This mixture was subjected to reflux for 30 minutes, filtered, and the resulting filtrate was transferred into a 100 mL volumetric flask. Subsequently, the residue underwent another reflux with 20 mL of acetone for 30 minutes, followed by filtration and combination with the volumetric flask, which was then augmented with acetone to achieve a total volume of 100 mL. A further step involved transferring 20 mL of the filtrate to a separating funnel, where it was combined with 20 mL of water. This mixture underwent thrice-extraction with 15 mL portions of ethyl acetate each time. The collected ethyl acetate fraction was then amalgamated with additional ethyl acetate in the volumetric flask to attain a total volume of 50 mL. Blank solution was meticulously concocted by drawing 10 mL of the stock solution and adding glacial acetic acid solution until the total volume reached 25 mL in the volumetric flask. For the sample solution, 10 mL of the stock solution was combined with 1 mL of AlCl_3_ solution and glacial acetic acid solution, leading to a total volume of 25 mL within the volumetric flask. After the introduction of AlCl_3_, measurements were conducted utilizing a spectrophotometer set at a wavelength of 425 nm, employing quercetine as the reference. These measurements were carried out after a 30-minute interval [24].

### 2.9. Determination of Total Phenolics

The procedure for determining the total phenol content was carried out as follows: for the standard (gallic acid), a gallic acid stock solution was prepared at a concentration of 5000 ppm. This was achieved by weighing 12.5 mg of gallic acid and dissolving it in analytical grade methanol within a 25 mL volumetric flask. Subsequently, a series of standard concentrations spanning 0, 10, 30, 50, 70, and 100 ppm were established within separate 25 mL flasks. From each concentration, 1 mL was pipetted into a test tube, followed by the addition of 1 mL of the gallic acid solution and 5 mL of the 7.5% Folin–Ciocalteu reagent. The mixture was meticulously mixed and left to incubate in a dark environment for approximately 8 minutes. Afterwards, 4 mL of the 1% NaOH reagent was introduced, mixed once more, and left to incubate for 1 hour in the dark. The solution was eventually assessed using a spectrophotometer, measuring at a wavelength of 730 nm. For the sample analysis, 10 mg of extract was precisely weighed and dissolved in a 25 mL volumetric flask using analytical grade methanol. Following this, 1 mL of the sample solution was drawn into a test tube, and 5 mL of the 7.5% Folin–Ciocalteu reagent was added, mixed, and allowed to incubate in the dark for around 8 minutes. Subsequently, 4 mL of the 1% NaOH reagent was introduced, mixed again, and incubated for 1 hour in a dark environment. The resulting solution was then evaluated using a spectrophotometer at a wavelength of 730 nm [25, 26].

### 2.10. Determination of Quercetine by HPLC

Quercetine quantification was conducted employing high-performance liquid chromatography (HPLC) to ascertain the quercetine content within the combined extracts. The analysis was executed utilizing a C18–250 × 4.60 mm, 5 *μ*m, 100 Å column, with a mobile phase composed of HPLC-grade potassium hydrogen phosphate (pH 2.4) and acetonitrile (75 : 25) in an isocratic fashion over a 30-minute period. The flow rate was consistently maintained at 1 ml/min, and the UV-visible detector was set to 340 nm [27, 28].

### 2.11. Statistical Analysis

IC_50_ values were ascertained from dose-inhibition (curve fit) analysis using nonlinear regression within GraphPad Prism Software, version 9 (Graph Pad Software, San Diego, CA, USA).

## 3. Results

### 3.1. In Vitro and In Vivo Antidiabetic Activity

**Figure 1:**
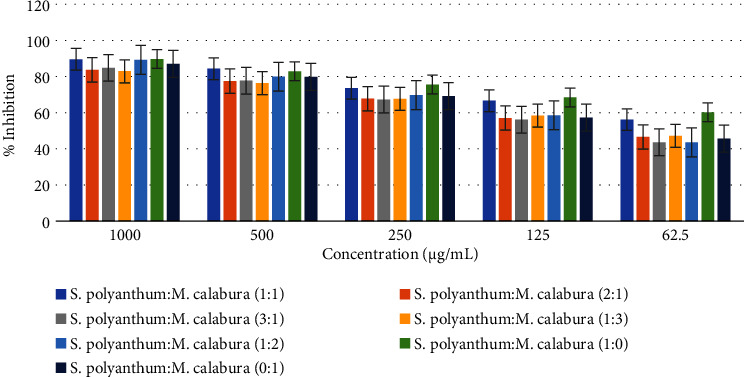
*In vitro* antidiabetic activity assay through inhibition of alpha-glucosidase enzyme activity.

In this study, we investigated the potential synergistic effects of individual and combined extracts of *S. polyanthum* and *M. calabura*, both of which are recognized for their antidiabetic properties. The primary objective was to evaluate their inhibitory activity against the *α*-glucosidase enzyme, thereby assessing the potential to enhance inhibition capacity through synergistic interactions. The results, as depicted in Figure 1, exhibited a dose-dependent profile of inhibition. Notably, increasing concentrations of the combined extracts correlated with higher percentages of inhibition. Remarkably, the inhibition rate observed in *S. polyanthum* extract demonstrated a lower IC_50_ value of 24.93 *µ*g/mL compared to that of *M. calabura* extract (81.05 *µ*g/mL). This result aligns with the findings observed when the extracts were combined in a 1 : 1 ratio, showcasing a significant inhibition rate of over 50% at a relatively low dose (62.5 *µ*g/mL). Notably, this combination displayed the lowest IC_50_ value of 36.42 *µ*g/mL, with significant differences (*p* < 0.05) compared among other combinations. Although the IC_50_ value of this combination (1 : 1) is not yet stronger than that of acarbose (standard drug), nevertheless it underscores the potent inhibitory effect of this mixture, as demonstrated by the IC_50_ value falling below 50 *µ*g/mL. In contrast, other combinations of extracts exhibited IC_50_ values ranging from 73.94 to 89.67 *µ*g/mL, indicating a moderate inhibitory effect (above 50 *µ*g/mL), as detailed in Table 1.

Table 2 presents the results of assessing the efficacy of a combination of a 96% ethanol extract of *S. polyanthum* and *M. calabura* in inhibiting the *α*-glucosidase enzyme, utilizing the Combination Index (CI) as the analytical parameter. Initiating with a low ratio of extracts in combinations serves as a straightforward method to ascertain the impact of each extract on both efficacy and toxicity. These preliminary data are crucial for identifying the influence of individual extracts when combined, enabling the estimation of the optimal combination ratio. Both the *S. polyanthum* and *M. calabura* extracts individually exhibit commendable antidiabetic activity through *α*-glucosidase enzyme inhibition. The synergistic effect of combining the 96% ethanol extract of *S. polyanthum* and *M. calabura* in a 1 : 1 ratio is evident, as indicated by a lower IC_50_ value and a Combination Index (CI) of less than 1, compared to other combinations that exhibit antagonism, which is shown by a CI > 1. This finding suggests a synergistic effect, consistent with prior research indicating that combining plant extracts enhances *α*-glucosidase enzyme inhibition through synergism, a positive interaction exceeding the sum of individual substances. However, beyond synergism, other combinations exhibit antagonism effects. Antagonism occurs when the combined activity is lower than that of each individual extract.

**Figure 2:**
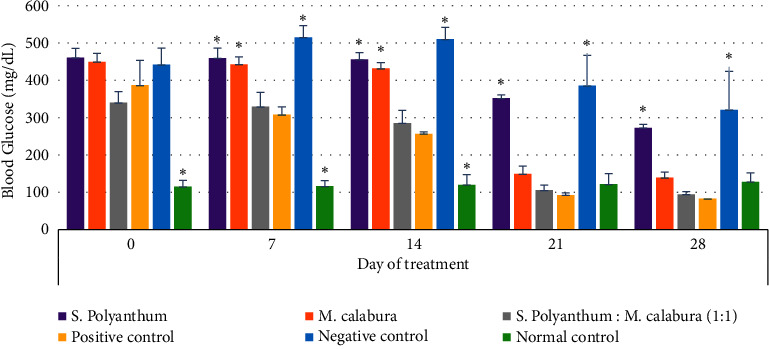
Effect of extracts on fasting blood glucose levels in normal control and streptozotocin-induced diabetic mice. *S. polyanthum* 200 mg/kg BW; *M. calabura* 200 mg/kg BW; extract combination 1 : 1 (200 mg/kg BW: 200 mg/kg BW); positive control glibenclamide (4.5 mg/kg BW). Data were expressed as mean ± SD (*n* = 5), ^*∗*^sign indicates significant difference between the treatment groups on each day at 95% confidence level (*p* < 0.05) using post hoc Tukey's HSD test.

The synergistic effect of a combined 1 : 1 ratio of *S. polyanthum* and *M. calabura* leaf extracts was investigated for its potential to ameliorate hyperglycemia in a streptozotocin-induced diabetic rat model. As depicted in Figure 2, the longitudinal assessment of blood glucose levels over a 28-day span revealed a consistent downward trend in the treatment group, in contrast to the negative control and normal control groups. Notably, glibenclamide, serving as a positive control, significantly attenuated blood glucose concentrations to 83 mg/dL throughout the duration of the treatment period.

The synergistic potential of a 1 : 1 combination of *S. polyanthum* and *M. calabura* leaves extract was further investigated for its efficacy in reducing blood sugar levels in streptozotocin-induced diabetic rats. According to Figure 2, the treatment group exhibited a consistent decrease in blood glucose levels over a 28-day period, in contrast to both the negative control and normal control groups. This outcome underscores the efficacy of the combined extracts in managing blood glucose levels. Notably, glibenclamide, employed as a positive control, significantly decreased blood glucose levels to 83 mg/dL, demonstrating a marked difference from the normal control (*p* < 0.005). Similarly, *M. calabura* extract exhibited superior efficacy in lowering blood glucose levels compared to *S. polyanthum* extract during the 28-day treatments. Surprisingly, when the two extracts were combined, their collective activity became more potent, resulting in a blood glucose level of 94 mg/dL, which is proved by a significant difference than the normal control (*p* < 0.005). Intriguingly, no significant differences were observed between the 1 : 1 extract combination and positive control glibenclamide, indicating the effectiveness of the combined dose as an antidiabetic agent. Furthermore, the extract combination demonstrated the highest average percentage decrease in blood glucose levels (72.35%), reinforcing the synergistic effect observed (Table 3).

### 3.2. Acute Oral Toxicity

**Figure 3:**
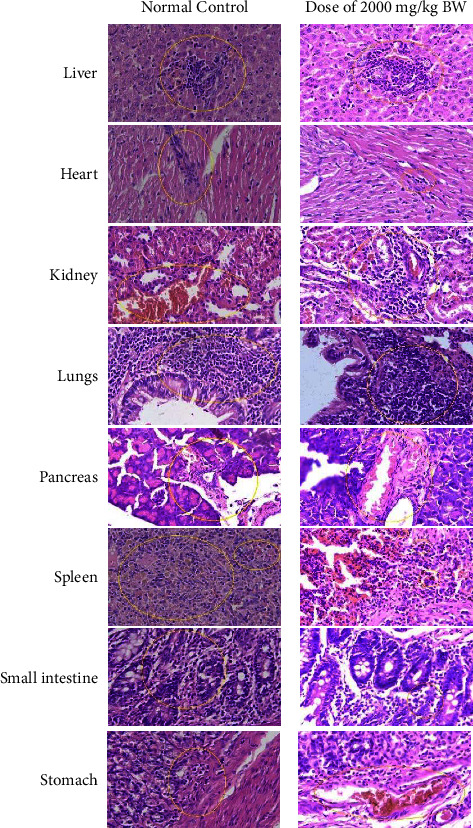
Histopathology of rat organs of 1 : 1 extract combination at a dose of 2000 mg/kg body weight (right) compared to normal control (left).

The acute toxicity study revealed that the combined administration of ethanolic extracts from *S. polyanthum* and *M. calabura* (1 : 1) exhibited no discernible signs of toxicity at doses up to 2000 mg/kg. This lack of toxicity was substantiated by the absence of significant alterations in various behavioral indicators, including alertness, motor activity, weight, sluggishness, paralysis, breathing, restlessness, diarrhea, convulsions, and coma. Furthermore, there were no fatalities observed over a two-week period, and the subjects remained physically active. The findings indicate that the tested doses of the plant extracts did not result in any observable adverse effects, suggesting that the median lethal dose (LD_50_) exceeds 2000 mg/kg body weight in rats. Consequently, the combined extract can be considered nontoxic, as its actual LD_50_ surpasses the 2000 mg/kg threshold. Additionally, a subsequent organ histopathology test at a dose of 2000 mg/kg body weight revealed mild changes and damage to the organs (Figure 3).

### 3.3. Total Phenolic, Total Flavonoid, and Quercetine Content

**Figure 4:**
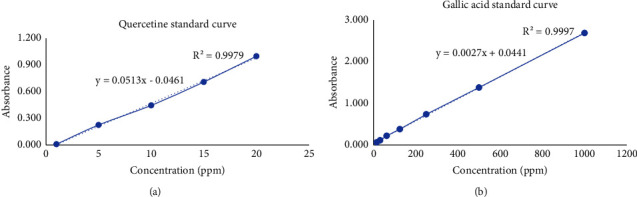
Linear regression curves of total flavonoids by using quercetine standard (a) and total phenolic by using gallic acid standard (b).

**Figure 5:**
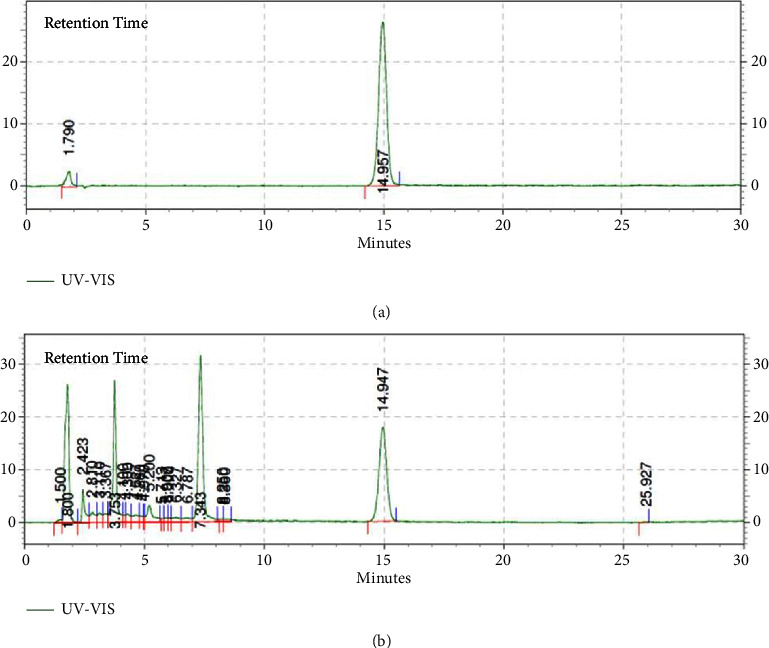
HPLC-chromatogram profile of quercetine standard (a) and 1 : 1 extract combination (b).

Table 4 presents the results for total phenolic and total phenolics. The determination of total phenolic content relied on the linear regression of the standard quercetine compound, following the equation *y* = 0.0513*x* − 0.0461 (*r*^2^ = 0.9979). The combined extract exhibited varying total phenolic content within the range of 24.51 to 32.40% w/w. Similarly, the quantification of total flavonoids was based on the linear regression of the gallic acid standard compound, with the equation *y* = 0.0027*x* − 0.0441 (*r*^2^ = 0.9997), as depicted in Figure 4. The total flavonoid content fell within the range of 0.67 to 1.37% w/w. Meanwhile, quercetine quantification showed that the combined extract (1 : 1) contained detectable levels of quercetine with a sharp peak that can be comparable with the quercetine standard at retention time of 14.947 minutes. The concentration of quercetine in the combined extract was found to be 3.25 mg/g (Figure 5).

## 4. Discussion

Patients with diabetes require meticulous management of their blood glucose levels, particularly during fasting and postprandial states. An enzyme known as *α*-glucosidase, predominantly present in the small intestine, plays a pivotal role in carbohydrate metabolism. Targeting this enzyme has emerged as a promising strategy for antidiabetic treatment. Inhibitors of *α*-glucosidase have demonstrated significant potential in managing postprandial hyperglycemia and treating diabetes by impeding the rapid conversion of complex carbohydrates into glucose. This inhibition leads to a controlled release of glucose into the bloodstream, thereby averting sudden spikes in blood glucose levels after meals, which is crucial for maintaining glycemic control in individuals with diabetes [29]. Several studies found that drugs capable of inhibiting the action of digestive enzymes like *α*-glucosidase can effectively regulate postprandial blood glucose levels in diabetic patients [30]. Moreover, studies have highlighted the inhibitory potential of various plant extracts on *α*-glucosidase, indicating their role in managing diabetes and postprandial hyperglycemia [31–33]. Additionally, natural compounds from plants or even the extracts and fractions have been investigated for their inhibitory effects on *α*-glucosidase, offering potential as alternative treatments for controlling high blood sugar levels [34, 35].

The exploration of natural products for antidiabetic properties has led to the identification of *S*. *polyanthum* and *M*. *calabura* as potential sources of antidiabetic agents. *S. polyanthum* and *M. calabura* are two medicinal plants that have been reported to possess antidiabetic properties based on an alpha-glucosidase inhibitory mechanism. The 50% ethanol extract of *M. calabura* and the 70% ethanol extract of *S. polyanthum* inhibited the enzyme with low IC_50_ values of 0.46 and 19.08 *µ*g/mL, respectively [16, 36]. In this work, the 96% ethanolic extract of *S. polyanthum* and *M. calabura* showed IC_50_ values of 24.39 and 81.05 *µ*g/mL, respectively. Interestingly, when the extracts were combined in a 1 : 1 ratio, the IC_50_ became 36.43 *µ*g/mL. The compounds with IC_50_ values below 50 *µ*g/mL are considered to have superior activity which made the combination of extracts from *S. polyanthum* and *M. calabura* show promising results in inhibiting key enzymes like *α*-glucosidase, which is essential in managing blood sugar levels in diabetes [37]. The low IC_50_ values observed in this combination suggest a higher potency in inhibiting these enzymes, potentially leading to better control of blood glucose levels. This phenomenon suggests a nuanced interaction between the compounds present in the two extracts when combined, leading to a modified bioactivity profile. This is substantiated by the fact in this study that reveal the combination manifested a synergistic effect, as evidenced by a Combination Index (CI) value of 0.94, which is less than 1. The concept of combining extracts to achieve a modified or enhanced biological effect or mitigate adverse effects. For instance, the combination of extracts has been shown to potentially offer synergistic effects, where the combined activity of the extracts is greater than or equal to the sum of their individual activities. Synergistic interactions can lead to enhanced bioactivity, which might result in a more potent therapeutic effect than would be expected from the individual activities of the single compounds alone [38–40]. Combining plant extracts has been shown to enhance the pharmacological activity potential due to the presence of multiple compounds, leading to synergistic or additive effects. This approach not only diversifies the sources of active ingredients but also reduces the pressure on specific plant species, supporting sustainability [41].

Further *in vivo* analysis showed that the combination of *S. polyanthum* and *M. calabura* extracts in a 1 : 1 ratio at a dose of 200 mg/kg BW demonstrated significant synergistic effects in reducing blood glucose levels. This synergism was evidenced by a remarkable 72.35% reduction in blood glucose levels after 28 days of treatment, surpassing the effects of the individual extracts of *S. polyanthum* and *M. calabura* alone. Another study demonstrated that the methanolic extract of *S. polyanthum*, at doses of 250, 500, and 1000 mg/kg BW, could reduce blood glucose levels in streptozotocin-induced rats. Additionally, the water extract of *M. calabura*, at a dose of 400 mg/kg BW, also reduced blood glucose levels and increased insulin selectivity [15, 42].

The 1 : 1 extract combination exhibited a notably low IC_50_ value in the inhibition of alpha-glucosidase and *in vivo* antidiabetic activity, a result that could be attributed to its elevated total flavonoid content. This higher flavonoid content likely played a pivotal role in conferring a strong category of alpha-glucosidase inhibitory and *in vivo* antidiabetic activity. Several reports have supported our results, showing that *M. calabura* contains several antidiabetic compounds derived from flavonoids and flavonoid glycosides such as geniposide, daidzein, quercitrin, 6-hydroxyflavanone, kaempferol, and formononetin. Meanwhile, *S. polyanthum* also contains antidiabetic compounds with flavonoids' type, including myricetin-3-O-rhamnoside (myricitrin) and epigallocatechin-3-gallate (EGCG) [16, 43]. Therefore, in this study, we propose quercetine, a flavonoid, as a bioactive and marker compound. Its presence has been identified in the extract combination (1 : 1) and exhibited a direct correlation with total flavonoid content, *in vitro* alpha-glucosidase inhibitory activity, and *in vivo* antidiabetic activity. Quercetine itself has been reported as a potent *α*-glucosidase inhibitor, demonstrating a competitive mechanism [44, 45]. These findings highlighted the potential for developing an extract combination of these two plants as a raw material for antidiabetic herbal medicine, particularly noteworthy for its demonstrated lack of toxicity in acute oral toxicity tests.

## 5. Conclusion

The synergistic potential of the combined ethanolic extracts from *M. calabura* and *S. polyanthum* has been proven to yield a significant *α*-glucosidase inhibitory and blood glucose level decreasing effect. Particularly, the 1 : 1 combination displayed the most enzymatic inhibitory activity, with an IC_50_ value of 36.43 mg/L and 73.25% of DGBL on streptozotocin-induced rats animal. This efficacy was found to correlate with the elevated concentrations of total phenolics, total flavonoids, and quercetine, which measured at 30.81% w/w, 1.37% w/w, and 3.25 mg/g, respectively. These findings highlighted the promising role of these combined extracts in the observed effects.

## Figures and Tables

**Table 1:** IC_50_ value of alpha-glucosidase inhibition of a combined ethanolic extract of *S. polyanthum* and *M. calabura*.

*S. polyanthum* : *M. calabura*	IC_50_ (*µ*g/mL)
1 : 1	36.43^a^
2 : 1	78.54^b^
3 : 1	89.67^c^
1 : 3	73.94^d^
1 : 2	84.77^e^
1 : 0	24.39^f^
0 : 1	81.05^g^
Acarbose	0.13^h^

The different subscript letters in the table indicate a significant difference between the treatment groups with a 95% confidence level (*p* < 0.05) using post hoc Tukey's HSD test.

**Table 2:** The combination index of alpha-glucosidase inhibition of the combined ethanolic extract of *S. polyanthum* and *M. calabura*.

*S. polyanthum* : *M. calabura*	CI values	Categories
1 : 1	0.94483	Synergism
2 : 1	1.79502	Antagonism
3 : 1	2.16043	Antagonism
t1 : 3	1.19171	Antagonism
1 : 2	1.51514	Antagonism

**Table 3:** Average percentage decrease of blood glucose level (% DBGL).

Treatment groups	(% DBGL)
*S. polyanthum* 200 mg/kg BW	40.78^a^
*M. calabura* 200 mg/kb BW	69.04^b^
*S. polyanthum*: *M. calabura* (1 : 1)	72.35^c^
Glibenclamide (4.5 mg/kg BW)	78.55^c^

The different subscript letters in the table indicate a significant difference between the treatment groups with a 95% confidence level (*p* < 0.05) using post hoc Tukey's HSD test.

**Table 4:** Total phenolic, total flavonoids, and quercetine levels of the combined extract of *S. polyanthum* and *M. calabura* leaves.

Combination extract (*S. polyanthum* : *M. calabura*)	Total phenolics (%w/w)	Total flavonoid (%w/w)	Total quercetine (%w/w)
1 : 1	30.81^a^	1.37^a^	3.25
2 : 1	24.51^b^	1.07^b^	1.68
3 : 1	32.40^c^	0.89^c^	3.59
1 : 3	32.37^c^	0.94^d^	8.06
1 : 2	31.40^d^	0.67^e^	5.65
1 : 0	29.50^e^	0.57^f^	0.52
0 : 1	19.76^f^	0.81^g^	8.44

The different subscript letters in the table indicate a significant difference between the treatment groups with a 95% confidence level (*p* < 0.05) using post hoc Tukey's HSD test.

## Data Availability

The datasets used in this study are available upon reasonable request to the corresponding author.

## References

[1] Saeedi P., Petersohn I., Salpea P. (2019). Global and regional diabetes prevalence estimates for 2019 and projections for 2030 and 2045: results from the international diabetes federation diabetes atlas, 9th edition. *Diabetes Research and Clinical Practice*.

[2] Chaturvedi R., Desai C., Patel P., Shah A., Dikshit R. (2018). An evaluation of the impact of antidiabetic medication on treatment satisfaction and quality of life in patients of diabetes mellitus. *Perspectives in Clinical Research*.

[3] Rao B. N. (2003). Bioactive phytochemicals in Indian foods and their potential in health promotion and disease prevention. *Asia Pacific Journal of Clinical Nutrition*.

[4] Benalla W., Bellahcen S., Bnouham M. (2010). Antidiabetic medicinal plants as a source of alpha glucosidase inhibitors. *Cancer Drug Resistance*.

[5] Grover J. K., Yadav S., Vats V. (2002). Medicinal plants of India with anti-diabetic potential. *Journal of Ethnopharmacology*.

[6] Dirir A. M., Daou M., Yousef A. F., Yousef L. F. (2022). A review of alpha-glucosidase inhibitors from plants as potential candidates for the treatment of type-2 diabetes. *Phytochemistry Reviews*.

[7] Danuri H. M., Lestari W. A., Sugiman U., Faridah D. N. (2020). In vitro *α*-glucosidase inhibition and antioxidant activity of mulberry (morus alba L.) leaf ethanolic extract. *Jurnal Gizi dan PanganAlba L. Leaf Ethanolic Extract. Jgizipangan.*.

[8] Moukette B. M., Ama Moor V. J., Biapa Nya C. P. (2017). Antioxidant and synergistic antidiabetic activities of a three-plant preparation used in Cameroon folk medicine. *International Scholarly Research Notices*.

[9] Perumal N., Nallappan M., Shohaimi S., Kassim N. K., Tee T. T., Cheah Y. H. (2022). Synergistic antidiabetic activity of *Taraxacum officinale* (L.) Weber ex F.H.Wigg and Momordica charantia L. polyherbal combination. *Biomedicine and Pharmacotherapy*.

[10] Septiana E., Rizka N. M., Yadi Y., Simanjuntak P. (2021). Antidiabetic activity of extract combination of Orthosiphon aristatus and Oryza sativa L. Var glutinosa. *Borneo Journal of Pharmacy*.

[11] Hasan A. E. Z., Andrianto D., Rosyidah R. A. (2022). Uji penghambatan *α*-glukosidase Dari kombinasi ekstrak kunyit, the hitam dan jahe: the *α*-glucosidase inhibition test from a combination of turmeric extract, black tea, and ginger. *European Journal of Bioethics*.

[12] Widiastuti T. C., Rahayu T. P., Lestari A., Kinanti A. P. (2023). Uji aktivitas antidiabetes kombinasi ekstrak terstandar daun salam (syzigium polyanthum walp.) dan daun ganitri (Elaeocarpus ganitri roxb.) pada tikus putih (*Rattus norvegicus*) jantan galur wistar yang diinduksi streptozotosin. *Asian Journal of Pharmaceutical and Clinical Research*.

[13] Widharna R., Ferawati T. W., Hendriati L., Hamid I., Widjajakusuma E., Widjajakusuma E. (2015). Antidiabetic effect of the aqueous extract mixture of andrographis paniculata and Syzygium polyanthum leaf. *European Journal of Medicinal Plants*.

[14] Setyawati A., Hirabayashi K., Yamauchi K. (2018). Melanogenesis inhibitory activity of components from Salam leaf (Syzygium polyanthum) extract. *Journal of Natural Medicines*.

[15] Aligita W., Susilawati E., Sukmawati I. K., Holidayanti L., Riswanti J. (2018). Antidiabetic activities of Muntingia calabura L. Leaves water extract in type 2 diabetes mellitus animal models. *The Indonesian Biomedical Journal*.

[16] Zolkeflee N. K. Z., Ramli N. S., Azlan A., Abas F. (2022). In vitro anti-diabetic activities and UHPLC-ESI-MS/MS profile of *Muntingia calabura* leaves extract. *Molecules*.

[17] Mahmood N. D., Nasir N. L. M., Rofiee M. S. (2014). *Muntingia calabura*: a review of its traditional uses, chemical properties, and pharmacological observations: a review of its traditional uses, chemical properties, and pharmacological observations. *Pharmaceutical Biology*.

[18] Widodo A., Sulastri E., Ihwan I., Mutiara M., Maulana S., Zubair M. S. (2024). Prospective utilization of combination of Salam (Syzygium polyanthum) and Kersen (*Muntingia calabura*) Leaves as natural antidiabetic: an in silico study. *Journal of Experimental Biology and Agricultural Sciences*.

[19] Sancheti S., Sancheti S., Seo S. Y. (2009). Chaenomeles sinensis: a potent *α*-and *β*-glucosidase inhibitor. *American Journal of Pharmacology and Toxicology*.

[20] Chou T., Martin N. (2005). *CompuSyn for Drug Combinations: PC Software and User’s Guide: A Computer Program for Quantitation of Synergism and Antagonism in Drug Combinations, and the Determination of IC50 and ED50 and LD50 Values*.

[21] Huang L., Jiang Y., Chen Y. (2017). Predicting drug combination index and simulating the network-regulation dynamics by mathematical modeling of drug-targeted EGFR-ERK signaling pathway. *Scientific Reports*.

[22] OECD (2001). Guideline for testing of chemicals-acute oral toxicity-up-and-down procedure. https://www2.oecd.org/chemicalsafety/test-no-425-acute-oral-toxicity-up-and-down-procedure-9789264071049-en.

[23] Tafesse T. B., Hymete A., Mekonnen Y., Tadesse M. (2017). Antidiabetic activity and phytochemical screening of extracts of the leaves of Ajuga remota Benth on alloxan-induced diabetic mice. *BMC Complementary and Alternative Medicine*.

[24] Suparno O., Panandita T., Afifah A., Marimin P. R., Purnawati R. (2018). Antibacterial activities of leave extracts as bactericides for soaking of skin or hide. *IOP Conference Series: Earth and Environmental Science*.

[25] Ainsworth E. A., Gillespie K. M. (2007). Estimation of total phenolic content and other oxidation substrates in plant tissues using Folin–Ciocalteu reagent. *Nature Protocols*.

[26] Sulastri E., Zubair M. S., Anas N. I. (2018). Total phenolic, total flavonoid, quercetin content and antioxidant activity of standardized extract of moringa oleifera leaf from regions with different elevation. *The Pharmaceutical Journal*.

[27] Geodakyan S. V., Voskoboinikova I. V., Kolesnik J. A., Tjukavkina N. A., Vasiliy L. I., Glyzin V. I. (1992). High-performance liquid chromatographic method for the determination of mangiferin, likviritin and dihydroquercetin in rat plasma and urine. *Journal of Chromatography B: Biomedical Sciences and Applications*.

[28] Yue-ling M., Yu-jie C., Ding-rong W., Ping C., Ran X. (2017). HPLC determination of quercetin in three plant drugs from genus sedum and conjecture of the best harvest time. *The Pharmaceutical Journal*.

[29] Iyswarya S., Visweswaran S., Muthukumar N. J., Tamilselvi S., Mathukumar S. (2022). Revealing Anti-diabetic potential of Siddha formulation Gandhaka Sarkkarai using alpha amylase and alpha glucosidase enzyme inhibition assay. *International Journal of Ayurvedic Medicine*.

[30] Jia Y., Ma Y., Cheng G., Zhang Y., Cai S. (2019). Comparative study of dietary flavonoids with different structures as *α*-glucosidase inhibitors and insulin sensitizers. *Journal of Agricultural and Food Chemistry*.

[31] Bhatia A., Singh B., Arora R., Arora S. (2019). In vitro evaluation of the *α*-glucosidase inhibitory potential of methanolic extracts of traditionally used antidiabetic plants. *BMC Complementary and Alternative Medicine*.

[32] Olaokun O. O., Zubair M. S. (2023). Antidiabetic activity, molecular docking, and ADMET properties of compounds isolated from bioactive ethyl acetate fraction of Ficus lutea leaf extract. *Molecules*.

[33] Olaokun O. O., Manonga S. A., Zubair M. S., Maulana S., Mkolo N. M. (2022). Molecular docking and molecular dynamics studies of antidiabetic phenolic compound isolated from leaf extract of englerophytum magalismontanum (sond.) T.D.penn. *Molecules*.

[34] Xu X., Lu F., Yang Z., Yan X., Akunne T., Li D. (2020). Comparative study on the antidiabetic efficacy of unripe and ripe fruit extracts of siraitia grosvenorii and the possible mechanism of action. *European Journal of Medicinal Plants*.

[35] Karigidi K. O., Olaiya C. O. (2020). Antidiabetic activity of corn steep liquor extract of Curculigo pilosa and its solvent fractions in streptozotocin-induced diabetic rats. *Journal of Traditional and Complementary Medicine*.

[36] Elya B., Handayani R., Sauriasari R. (2015). Antidiabetic activity and phytochemical screening of extracts from Indonesian plants by inhibition of alpha amylase, alpha glucosidase and dipeptidyl peptidase IV. *Pakistan Journal of Biological Sciences*.

[37] Assefa S. T., Yang E. Y., Chae S. Y. (2019). Alpha glucosidase inhibitory activities of plants with focus on common vegetables. *Plants*.

[38] Jin D., He J., Zhang K., Luo X., Zhang T. (2021). *α*‐Glucosidase inhibition action of major flavonoids identified from *Hypericum attenuatum* choisy and their synergistic effects. *Chemistry and Biodiversity*.

[39] Usman H. (2023). Glyceamic modulatory effects of a recipe from some commonly consumed fruit pulps in normal and streptozotocin-induced diabetic rats. *Arid-Zone Journal of Basic and Applied Research*.

[40] Bursal E., Aras A., Kılıç Ö, Taslimi P., Gören A. C., Gülçin İ (2019). Phytochemical content, antioxidant activity, and enzyme inhibition effect of *Salvia eriophora* Boiss. and Kotschy against acetylcholinesterase, *α*-amylase, butyrylcholinesterase, and *α*-glycosidase enzymes. *Journal of Food Biochemistry*.

[41] Seepe H. A., Nxumalo W., Amoo S. O. (2021). Natural products from medicinal plants against phytopathogenic Fusarium species: current research endeavours, challenges and prospects. *Molecules*.

[42] Widyawati T., Yusoff N., Asmawi M., Ahmad M. (2015). Antihyperglycemic effect of methanol extract of Syzygium polyanthum (wight.) leaf in streptozotocin-induced diabetic rats. *Nutrients*.

[43] Syabana M. A., Yuliana N. D., Batubara I., Fardiaz D. (2022). *α*-glucosidase inhibitors from Syzygium polyanthum (Wight) Walp leaves as revealed by metabolomics and in silico approaches. *Journal of Ethnopharmacology*.

[44] Proença C., Freitas M., Ribeiro D. (2017). *α*-Glucosidase inhibition by flavonoids: an in vitro and in silico structure-activity relationship study. *Journal of Enzyme Inhibition and Medicinal Chemistry*.

[45] Shen H., Wang J., Ao J. (2022). Inhibitory kinetics and mechanism of active compounds in green walnut husk against *α*-glucosidase: spectroscopy and molecular docking analyses. *Lebensmittel-Wissenschaft und-Technologie*.

